# Unexpected increase in the bone marrow toxicity of mitomycin C (MMC)

**DOI:** 10.1054/bjoc.2000.1644

**Published:** 2001-03

**Authors:** Masataka Yoshimoto, Mitsue Saito, Takashi Tada, Kaoru Takahashi, Fujio Kasumi

**Affiliations:** Breast Surgery Department, Cancer Institute Hospital, Kami-Ikebukuro 1-37-1 Toshima-ku, Tokyo, 170-8455, Japan


*British Journal of Cancer* (2001) **84**(5), 736–737
© 2001 Cancer Research Campaign
doi: 10.1054/ bjoc.2000.1644, available online at http://www.idealibrary.com on IDEAL

Letter to the Editor

### Unexpected increase in the bone marrow toxicity of mitomycin C (MMC)

**Sir**

MMC, a product of *Streptomyces caespitosus*, is known to be one of the most active agents for various neoplasms and has been widely used throughout the world for over 40 years (Wakaki et al, 1958; Hortobagyi, 1985). Recently, however, we have noticed an unexpected increase in MMC's toxicity. Although the reasons for this are under investigation, we feel it necessary to draw this matter to the attention of clinicians who prescribe the drug.

From 1990 to 1996, we performed a prospective randomized clinical trial comparing the effects of CMF versus MMC plus CMF (MCMF) in the treatment of node-positive, premenopausal breast cancer patients (age ≤ 50) in an adjuvant setting after obtained informed consent. In the MCMF group, the dose and time schedule of MMC was 14 mg/m$^2$ on day 1 immediately after surgery, and 8 mg/m$^2$ on day 2, and 6 cycles of CMF chemotherapy were started after recovery from the toxicity of MMC, usually after one month. Total enrolled number of patients was 283. The main toxic effect of MMC monotherapy was bone marrow suppression, but patients were well tolerated during the course of the trial. After a median follow-up of 5.7 years, a statistically significant advantage of MMC plus CMF over CMF alone in relapse-free survival was observed ($P$ = 0.0058), but not in overall survival (the 36th ASCO #364). After the end of the trial, we adopted a MCMF regimen as a standard therapy for patients in the same condition in view of the favorable results obtained. However, this time we noticed a gradual, and unexpected but significant increase of MMC related bone marrow toxicity from about 1995 or 1996 (Figure 1).

As the change in toxicity was unequivocal, we tried to clarify its causes. In a survey of possible dependent factors, no apparent differences were revealed regarding the distribution of patient age, disease stage, actually administered dose, or time schedule of administration between patients who participated in the trial and recent non-trial patients, or between patients who developed grade III/IV toxicity and those who did not. Following this, another two possible causes were explored. First, we tested the consistency of the quality of MMC itself against various lots of products physically and chemically using spectrography (HPLC), chromatography, net weight deviation, and bioassay of MMC's power against bacteria, but we found no differences. The company (Kyowa Hakko Kogyo Co) carried out an in vivo animal toxicity test using BALB/C mice, but the results showed no differences in the bone marrow toxicity between product lots produced in 1994, 1997 and 1998. Secondly, since drugs other than cytostatics used concomitantly during the course of MMC treatment might affect its toxicity, these 82 drugs were surveyed. Several drugs including antiserotonin agents and an anesthetic were frequently used in the grade III/IV toxicity group, but no clear conclusions could be obtained yet. An in vivo animal toxicity test of MMC together with these potential drugs is now being carried out.

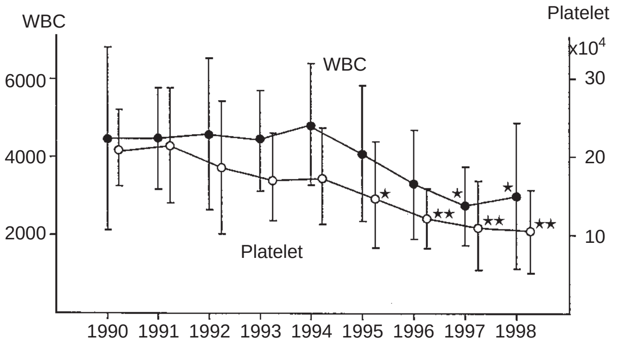

**Figure 1** Trends of the mean nadir levels of white blood cells and platelet counts each year following MMC treatment. A significant decrease of the mean nadir levels of white blood cell counts is seen in 1997–1998 and in platelets counts in 1995–1998 compared to those of 1990–1991, respectively (*: $P < 0.01$, **: $P < 0.0001$). Grade III/IV bone marrow toxicity was seen in 6% (8/135) of patients during the course of 1990–1996, whereas it was seen in 35% (15/43) during 1997–1998 ($P < 0.0001$). Patients who had previously been administered anti-cancer drugs and who had had diseases that might affect bone marrow function are excluded from the data

In conclusion, MMC should be used with caution whatever the reason for its increase in toxicity.

*Masataka Yoshimoto, Mitsue Saito, Takashi Tada, Kaoru Takahashi and Fujio Kasumi*
*Breast Surgery Department, Cancer Institute Hospital, Kami-Ikebukuro 1-37-1, Toshima-ku, Tokyo 170-8455, Japan*

### REFERENCES

Hortobagyi GN (1985) Mitomycin-C in breast cancer. *Semin Oncol* **12**: 65–70
Wakaki S, Marumo H, Tomioka K, et al (1958) Isolation of new fractions of antitumor mitomycins. *Antibiot Chemother* **8**: 228–240

